# In Vitro Anticandidal Activity and Mechanism of a Polyoxovanadate Functionalized by Zn-Fluconazole Complexes

**DOI:** 10.3390/molecules23051122

**Published:** 2018-05-09

**Authors:** Shuanli Guo, Wei Yang, Mingming Zhao, Rui Tian, Boyu Zhang, Yanfei Qi

**Affiliations:** 1 School of Public Health, Jilin University, Changchun 130021, China; guosl15@mails.jlu.edu.cn (S.G.); mingming17@mails.jlu.edu.cn (M.Z.); tianrui16@mails.jlu.edu.cn (R.T.); boyu17@mails.jlu.edu.cn (B.Z.); 2 College of Basic Medical Science, Jilin University, Changchun 130021, China; ywei@jlu.edu.cn

**Keywords:** polyoxovanadate, fluconazole, antifungal activity, ergosterol, rt-PCR

## Abstract

The rise in the number of fungal infections is requiring the rapid development of novel antifungal agents. A new polyoxovanadate functionalized by Zn-fluconazole coordination complexes, Zn_3_(FLC)_6_V_10_O_28_·10H_2_O (ZnFLC) (FLC = fluconazole) has been synthesized and evaluated for in vitro antifungal against *Candida* species. The identity of ZnFLC were confirmed by elemental analysis, IR spectrum, and single-crystal X-ray diffraction. The antifungal activities of ZnFLC was screened in 19 *Candida* species strains using the microdilution checkerboard technique. The minimum inhibitory concentration (MIC_80_) value of ZnFLC is 4 μg/mL on the azole-resistant clinical isolates of *C. albicans* HL973, which is lower than the positive control, FLC. The mechanism of ZnFLC against *C. albicans* HL973 showed that ZnFLC damaged the fungal cell membrane and reduced the ergosterol content. The expression of ERG1, ERG7, ERG11 ERG27, and ERG28, which have effects on the synthesis of ergosterol, were all significantly upregulated by ZnFLC.

## 1. Introduction

The *Candida* species including *C. albicans*, *C. glabrata*, *C. parapsilosis*, *C. tropicalis*, and *C. krusei* are posing serious nosocomial threats to patient populations [1,2,3]. Especially, the incidence of invasive *Candida* infection increases significantly with the increasing number of immunocompromised syndrome (e.g., AIDS), organ transplant patients, and patients receiving chemotherapeutic agents for cancer treatment [4]. Meanwhile, one of the most important manifestations of a systemic candidiasis concerns the oral mucosa. In these cases, differential diagnosis and curing of malignant and premalignant conditions is a must [5]. Azoles—such as fluconazole, itraconazole, posaconazole, and voriconazole—are considered the first-line treatment of patients with *Candida* infections [6,7]. However, with the widespread and prolonged use of azoles to treat fungal infections, drug resistance has become an increasing problem in clinic isolates [7,8]. Therefore, there is an urgent need to develop novel efficient antifungals.

Polyoxovanadate, a unique class of metal-oxide clusters, have various properties that make them attractive for applications in catalysis [9], magnetic [10,11], functional materials [12,13], and medicine [14,15,16,17]. The orange decavanadate that contains 10 vanadium atoms are the predominant polyoxovanadate species in the acidic pH range have attracted attention due to their medicinal and biochemical behavior [14,17]. In the neutral pH range, it is kinetically inert and remains in solution for several days. Decavanadate impacts proteins, lipid structures, and cellular function, and show some in vivo effects on oxidative stress processes [15]. Moreover, decavanadate were found to form inside yeast [18]. However, to our knowledge, antifungal evaluation of decavanadate remains scarcely reported by far. 

Ergosterol is an important component throughout the fungal cell membranes, which distinguishes fungi from bacteria, plant, and animal cells. It plays a vital role in many biological functions such as maintaining cell integrity, regulating membrane fluidity, and the cell cycle. Ergosterol biosynthesis pathway is thus a significant target of most existing antifungals and new antifungals under development, for instance, fluconazole, itraconazole, amphotericin B, terbinafine, etc. [19,20]. Based on our previous studies, the fungal membrane is the target of polyoxotungstates [21], and the antibacterial polyoxotungstates uptake in the cell were preferentially located on the membrane with intact composition [22,23]. Some examples have been reported that the pumps, channels, metalbotropic receptors, lipid structures are all the potential biological targets for decavanadate [14]. Therefore, it is necessary to research the interactions of polyoxovanadates on the membranes target. 

We and other groups recently demonstrated that polyoxotungstates have antifungal activities against various *Candida* species and agricultural fungal pathogens [21,22,23]. As a continuing work, we synthesized a new compound, Zn_3_(FLC)_6_V_10_O_28_·10H_2_O (ZnFLC) which consists of the decavanadate, zinc, and fluconazole. We evaluated the antifungal effect of ZnFLC against different species of *Candida* fungus. To investigate the antifungal mechanism of ZnFLC, the ergosterol contents were determined by high performance liquid chromatography (HPLC), and real-time PCR. This study demonstrates that ZnFLC is a potential antifungal candidate against the *Candida* species.

## 2. Results and Discussion

### 2.1. Structure Description of ZnFLC

**Figure 1:**
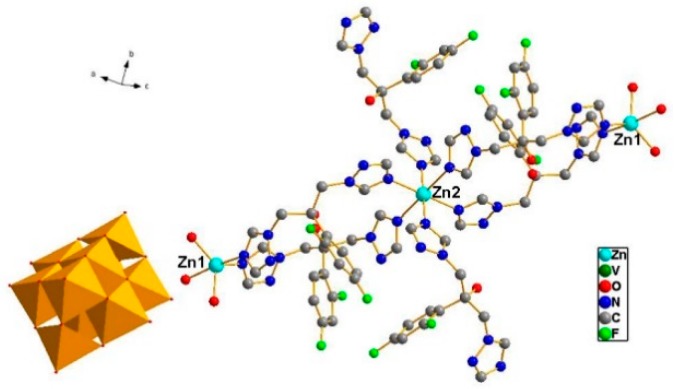
Combined ball-stick and polyhedral representation of **1**. The VO_6_octahedra are shown in green and the balls represent Zinc (light blue), carbon (black), nitrogen (blue), and oxygen (red).

Single crystal X-ray crystallography shows that compound ZnFLC, Zn_3_(FLC)_6_V_10_O_28_·10H_2_O, consists of [V_10_O_28_]^6−^ anions, [Zn_3_(FLC)_6_]^6+^ and the isolated H_2_O molecules (Table 1). In the asymmetric unit, there are two Zn(II) cations, three FLC ligands, and one [V_10_O_28_]^6−^ anion. As shown in Figure 1, the decavanadate [V_10_O_28_]^6−^ anion shows γ-isomer, which is composed of 10 edge-sharing VO_6_octahedra. There are five crystallographically independent V atoms in the unit. The V-O distances of each MoO_6_ octahedron can be divided into three groups: V-O_t_ (terminal) 1.597(5)–1.702(4) Å, V-O_12_ (central) 2.086(4)–2.325(4) Å, and V-O_b_ (bridge) 1.687(4)–2.029(4) Å. The bond valence sum calculations give the values of all vanadium sites are in the 5+ oxidation state. As shown in Figure S1a, the Zn(1) cation is coordinated by two nitrogen atoms [Zn(1) − N(1) = 2.021(6) Å and Zn(1) − N(13) = 2.122(6) Å] from different FLC molecules and three water molecules [Zn(1) − O(W1) = 2.019(5) Å, Zn(1) − O(W2) = 1.982(6) Å and Zn(1) − O(W3) = 2.180(6) Å], showing a trigonalbipyramidal coordination geometry [ZnN_2_O_3_]. The Zn(2) cation is six-coordinated by six nitrogen atoms from different FLC ligand with Zn(2) − N(10) = 2.129(6) Å, Zn(2) − N(10)#2 = 2.129(6) Å, Zn(2) − N(7)#2 = 2.220(6) Å, Zn(2) − N(7) = 2.220(6) Å, Zn(2) − N(6) = 2.277(6) Å, and Zn(2) − N(6)#2 = 2.277(6) Å, respectively (Figure S1b). It shows octahedral coordination geometries [ZnN_6_]. The Zn–O and Zn–N bond lengths are all within the normal ranges.

### 2.2. FT-IR of ZnFLC

The IR spectrum of ZnFLC has the characteristic asymmetric stretching vibration peaks at 956, 918, 815, 743, 670, 651, 631, 597, 515, and 456 cm^−1^, which are attributed to *ν*(V-O_terminal_) and *ν*(V-O_bridge_) of polyoxoanion, shown in Figure S2. The characteristic peaks of ZnFLC are nearly consistent with the reported decavanadate in the literature. The strong absorption bands at 3126, 1768, 1600, 1505, 1418, 1355, 1272, 1214, 1122, 1088, and 1039 are attributed to the characteristic peaks of the FLC ligands. The other strong features at 1617 cm^−1^ are assigned to the water molecules.

### 2.3. Antifungal Susceptibility Testing

The MICs of ZnFLC and FLC were evaluated for standard and clinical strains of *Candida* spp. As shown in Table 2, the MIC values of ZnFLC were various on different fungal strains. Except the clinical strains HL3084, HL17034, HL3970, and the standard strain ATCC 750, the activity of ZnFLC with MIC_50_ and MIC_80_ values of 0.5–64 μg/mL and 1–128 μg/mL were slightly higher than those of FLC. Because the FCZ-resistant clinic isolate HL973 was sensitive to ZnFLC, further studies were concentrated on the antifungal activities and the mechanism of ZnFLC against *C. albicans* HL973.

*Candida* species HL973, HL27, HL981, HL946, HL963, HL981, HL996, ATCC 750, ATCC 22019, and ATCC 90028 strains were treated with equivalent doses of Zn(oac)_2_, NaVO_3_ and FLC in ZnFLC (wt/wt %) at the MIC_80_ (4 μg/mL). As shown in Figure S3 the HL973, HL27, and HL963 strains in the medium group with or without Zn(oac)_2_ and NaVO_3_ had similar growth rates, indicating that Zn(oac)_2_ and NaVO_3_ had minimal effects. The FLC had inhibitory efficacy but lower than that of ZnFLC. The result indicated that the antifungal activities of ZnFLC are not from the simple mixture of metal cations and the FLC.

### 2.4. Inhibitory of ZnFLC on C. albicans HL973

**Figure 2:**
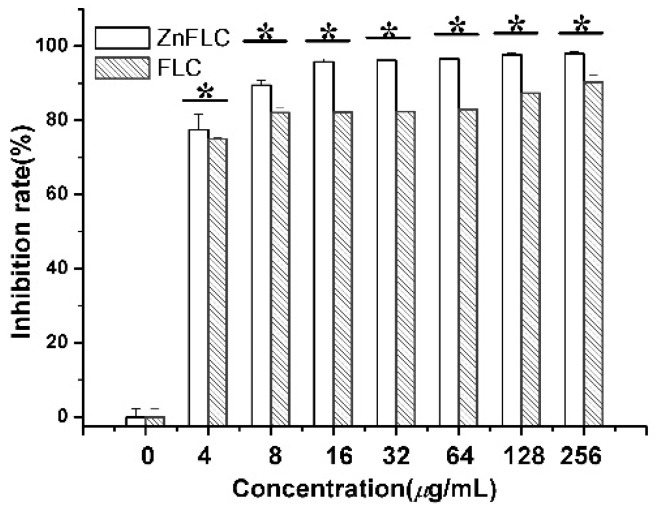
Inhibitory effect of ZnFLC and FLC on HL973 in different doses by MTX assay. Data are presented as the mean ± SD of three independent experiments. (* *p* < 0.05 for the ZnFLC or FLC vs. DMSO control.)

The inhibitory of the *C. albicans* HL973 cells in the presence of various concentrations of ZnFLC and FLC was evidenced once more by MTS method. As shown in Figure 2, after treatment by drugs, the viability of *C. albicans* HL973 cells of ZnFLC have a significant reduction than that in the negative control group (*p* < 0.05). The inhibition ratio of ZnFLC-treated *C. albicans* HL973 cells at 48 h with the concentrations of 4, 8, 16, 32, 64, and 128 μg/mL of ZnFLC reached the peak value 77.44, 89.43, 95.71, 96.10, 96.57, 97.64, and 97.89%, respectively. The antifungal activities of ZnFLC are dose-dependent. The result is similar to that from the broth microdilution method.

### 2.5. Growth Inhibition Curves

**Figure 3:**
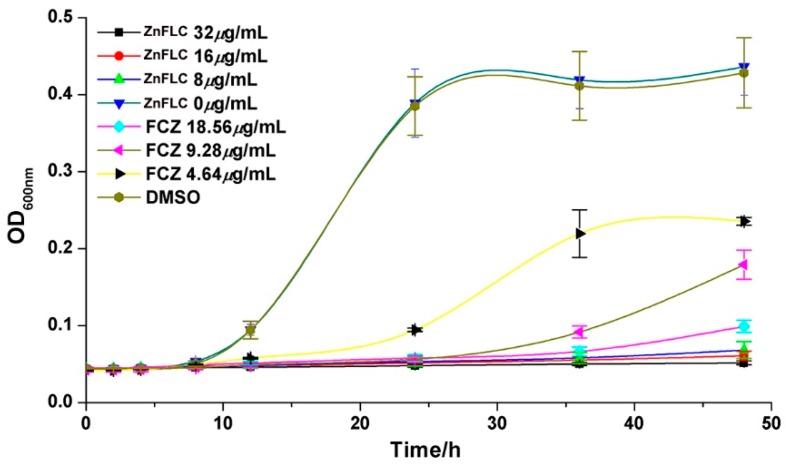
Inhibitory effect of ZnFLC and on HL973 in different time and different doses. Data are presented as the mean ± SD of three independent experiments.

The growth inhibition effect in the presence of various concentrations of ZnFLC, negative control, and FLC groups on the *C. albicans* HL973 at different times are given in Figure 3. HL973 strains in the medium group with or without DMSO had similar growth rates, indicating that DMSO had minimal effects. In comparison, the yeast treated with ZnFLC and FLC with the increasing inhibitor concentrations had significant delay in growth before 24 h. After 24 h treatment, the inhibited delay curve quickly increased in the 4.64 μg/mL FLC group. In comparison among the delay curves, the yeast receiving ZnFLC had a significantly higher inhibition rate than that in the FLC groups (*p* < 0.05).

### 2.6. Cell Living/Dead Analysis on HL973

**Figure 4:**
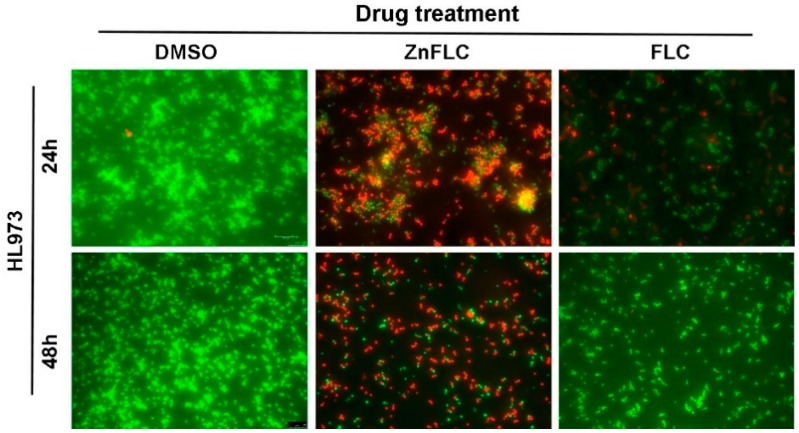
Fluorescent microscopy of HL973 cells treated with 64 μg/mL of ZnFLC and equivalent dose of FLC in ZnFLC (wt/wt %) at 24 h stained with AO (red) and EB (green). Scale bar: 30 μm.

The *C. albicans* HL973 were plated on 6-well plates at a density of 1.0 × 10^6^ cells/mL. After a 24 h or 48 h in period of incubation, the fluorescence microphotographs of HL973 cells treated with (0.5%) DMSO, ZnFLC, and FLC for 24 h and stained with AO/EB were shown in Figure 4. The presence of DMSO on *Candida* cells in applied concentrations (up to 0.5%) did not cause any adverse effects. In the presence of ZnFLC or FLC, large EB-positive areas (red staining) were evident, whereas the yeast cells were AO positive (green), indicating they were viable. ZnFLC was more effective at killing HL973 yeast cells than that of FLC.

### 2.7. Assessment of Ergosterol Content

**Figure 5:**
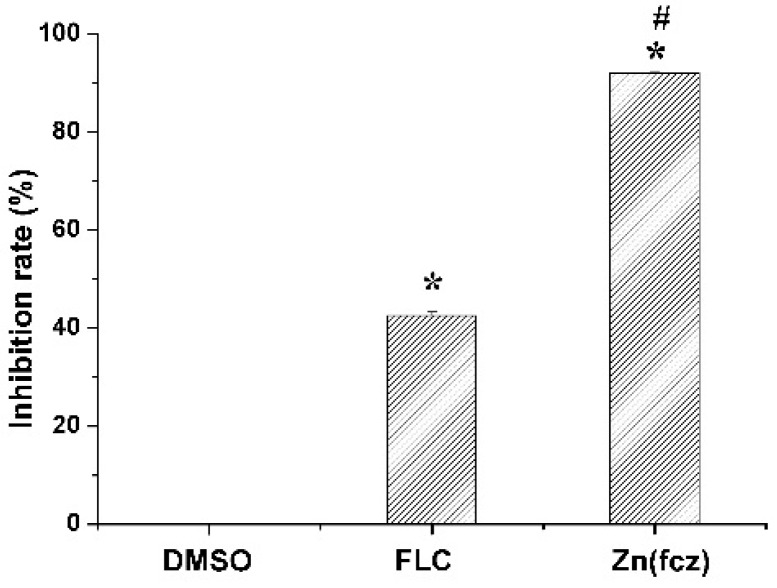
Concentration changes of ergosterol in *C. albicans* HL973 treated with 16 μg/mL of ZnFLC and equivalent dose of FLC in ZnFLC (wt/wt %) of FLC at 24 h using HPLC method. The experiment was performed in triplicate. Data were represented as mean ± SD. * *p* < 0.05 for the ZnFLC or FLC vs. DMSO control. ^#^ *p* < 0.05 for the ZnFLC vs. FLC at MIC.

In order to explore the reason of ZnFLC inhibiting the *C. albicans* HL973, the ergosterol contents, one kind of critical components in *C. albicans*, were determined by HPLC. The results showed that the retention time of ergosterol was about 12.90 min (Figure S4). After 24 h of treatment, the ergosterol contents in the control, FLC and ZnFLC extract were 7.46 ± 0.11, 4.29 ± 0.10, and 0.60 ± 0.03 mg/mL, respectively (*p* < 0.05) (Table S2). The standard curve was linear (*R*^2^ = 0.995). The treatment of *C. albicans* HL973 with ZnFLC and FLC resulted in a reduction of 92.02% and 42.53% of ergosterol content (*p* < 0.05), shown in Figure 5. The ergosterol content of the cytomembrane was obviously lower in the drug and FLC groups. The results indicated that one of ZnFLC inhibition the *C. albicans* HL973 had the similar was through inhibition of ergosterol biosynthesis.

### 2.8. The Level of Ergosterol Biosynthesis Related Genes

**Figure 6:**
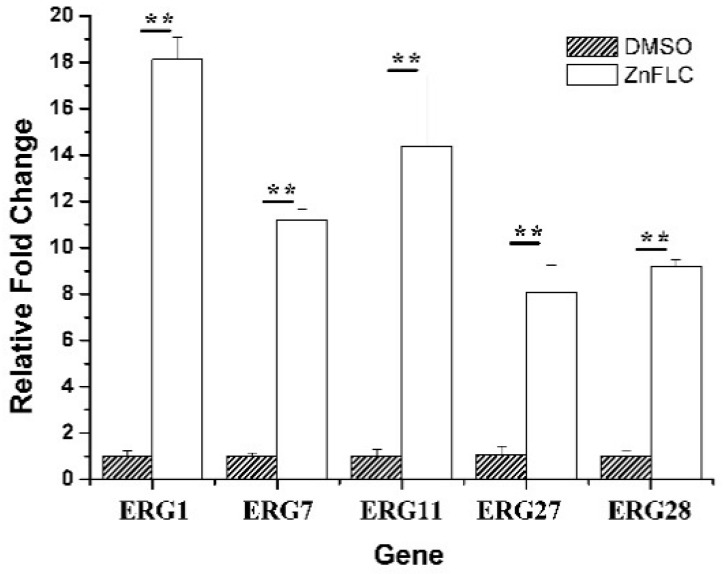
Expression of ERG genes is increased in clinical isolates HL973. RT-PCR was performed using RNA extracted from cells grown for 24 h treated with 2MIC_80_ ZnFLC. All data are normalized to an internal control and are expressed as fold induction relative to the expression level in strain. (** *p* < 0.01 for the ZnFLC vs. DMSO control.)

To further study the mechanism of ergosterol biosynthesis reduction by ZnFLC, real-time PCRs were used to evaluate the expression of five important genes involving in ergosterol biosynthesis. The *C. albicans* HL973 cells were exposed to ZnFLC at 8 μg/mL values for 24 h, their total RNA was extracted, and cDNA was synthesized by reverse transcription. This cDNA was then used as a template for a series of real-time PCRs. The results showed that the expression of *ERG1*, *ERG7*, *ERG11*, *ERG27*, and *ERG28* were significantly upregulated with the fold change relative to control of 18.11 ± 0.96, 11.19 ± 0.47, 14.39 ± 3.06, 8.07 ± 1.19, and 9.19 ± 0.28, respectively, as shown in Figure 6. These results is consistent with previous reports which *C. albicans* treated with azole [24,25]. The above results indicate that ZnFLC may inhibit the *C. albicans* growth at least partly through interfering the expression of ergosterol biosynthesis related genes, therefore decreasing the ergosterol contents and damaging the cell member of *C. albicans.* When sterol levels are reduced, the expression of ergosterol biosynthesis genes (*ERG*) are substantially increased. Therefore, we speculate that ZnFLC may inhibit the ERG genes expression and ergosterol biosynthesis, which is similar to the drugs reducing the sterol levels.

## 3. Materials and Methods

### 3.1. Chemicals and Machines

All the chemicals were analytical grade and used without further purification. RPMI-1640 medium (Sigma, Mendota Heights, MN, USA) buffered to pH 7.0 with MOPS (Sigma) was used for MIC determination and liquid culture of fungal strains. Fluconazole was purchased from TCI Company (Gurugram, India). Ergosterol standard was purchased from Dr. Ehrenstorfer Company (Augsburg, Germany). Prime script RT reagent kit (TaKaRa, Shiga, Japan) was used for reverse transcription. SYBR Green I (Roche, Basel, Switzerland) was used for real-time PCR reactions. IR spectrum was recorded in the range 400–4000 cm^−1^ on an Alpha Centaurt FT/IR Spectrophotometer using KBr pellets.

### 3.2. Synthesis and Characterization of Zn_3_(FLZ)_6_V_10_O_28_·10H_2_O

A mixture of Zn(OAc)_2_·2H_2_O (0.0465 g, 0.2 mmol), NaVO_3_ (0.045 g, 0.36 mmol), and FLC (0.03 g, 0.1 mmol) in water (7 mL) was stirred for 1 h. Then the mixture was placed in a 25 mL Teflon-lined autoclave and kept at 120 °C for six days. After the mixture was cooled to room temperature at 10 °C·h^−1^, orange crystals of ZnFLC were obtained in 68.9% yield based on Zn(OAc)_2_·2H_2_O. Elemental analyses calcd. for C_78_H_72_F_12_N_36_O_44_V_10_Zn_3_: C 29.70; H 2.28; N 15.99. Found: C 29.10; H 2.34; N 15.91. IR (KBr, cm^−1^): 3126(s), 1768(w), 1600(w), 1617(s), 1505(s), 1418(s), 1355, 1272(s), 1214(s), 1122(s), 1088(w), 1039(w), 986(s), 956(s), 918(s), 815(s), 743(vs), 670(s), 651(vs), 631(vs), 597(vs), 515(vs), and 456(vs).

### 3.3. X-ray Crystallography

The structure of ZnFLC was determined by single crystal X-ray diffraction. Data were collected on a Bruker D8 Venture diffractometer with Mo-Kα (*λ* = 0.71073 Å) at 300 K. Empirical absorption corrections (*φ* scan) were applied for ZnFLC. The structures were solved by the direct method and refined by the full-matrix least squares on *F*^2^ using the SHELXL-2003 software (version 6.14). All of the non-hydrogen atoms were refined anisotropically. Hydrogen atoms of organic ligands were fixed in ideal positions. The hydrogen atoms attached to water were not located. A summary of crystal data and structure refinements for ZnFLC is provided in Table 1. The selected bonds and angels are listed in Table S1. Crystallographic data for the structural analysis have been deposited with the Cambridge Crystallographic Data Center, CCDC reference number 1821286, for ZnFLC.

### 3.4. Fungal Isolates and Culture Conditions

The microorganisms used in this study consisted of 15 *Candida albicans*, 1 *Candida glabrata*, 1 *Candida krusei*, 1 *Candida parapsilosis*, and 1 *Cryptococcus tropicalis* strains. The strains named after HL were isolated from clinical fungal infection patients in Changchun Qian Wei hospital (China). The *Candida* species were preliminarily identified according to the colored colony morphology on CHROMagar *Candida* medium (CHROMagar Co., Paris, France) was used for the confirmatory identification of *Candida* species. The reference strains, *C. parapsilosis* ATCC 22019, *C. albicans* ATCC 90028, and *Candida tropicalis* ATCC 750 were obtained from American Type Culture Collection. All isolates were cultured at 35 °C and maintained on Sabouraud dextrose agar (SDA, Conda) at 4 °C in School of Public Health, Jilin University, China.

### 3.5. Determination of MIC of ZnFLC

The quality control strain, *C. parapsilosis* ATCC 22019 was included in each susceptibility test to ensure quality control. The results of MIC were determined by means of the broth microdilution method after incubation at 35 °C for 48 h. The minimal inhibitory concentration (MIC) values of ZnFLC and FLC were determined for all the *Candida* strains using Clinical and Laboratory Standards Institute (CLSI) broth microdilution method M38-A. Briefly, the fungal strains were cultured 18 h at 35 °C in SDB and suspended in 1640 medium to give a final density of 0.4~5 × 10^4^ cells/mL. The 96 well plates were prepared by dispensing into each well 100 μL of RPMI-1640 broth. A 100 μL of drugs initially prepared at the concentration of 512 μg/mL (1% DMSO) was added into each of the first wells, followed by two-fold serial dilution to obtain concentration range of 0.25~256 μg/mL. To this, 100 μL of 0.4~5 × 10^4^ cells/mL fungal cell suspensions was separately added. The 11th well contained 100 μL medium without drugs and fungal cell as the empty control. The last well contained 100 μL fungi cell suspensions without drugs as the negative control. The final volume in each well was 200 μL. The final concentration of DMSO in each medium was 1%, which did not affect the growth of the tests microorganisms. After agitation for 15 s, the plates were incubated at 35 °C for 48 h. The absorbance was measured at 600 nm on a microplate reader (Biotek Co., Winooski, VT, USA). MIC_80_ or MIC_50_ were determined as the lowest concentration of the drugs that inhibited growth by 80% or 50% compared with that of drug free wells. The inhibitory rate was calculated using the equation

$$\mathrm{Inhibitory}\;\mathrm{rate}\left(\%\right)=1-\frac{OD_{drug}-OD_{empty\;control}}{OD_{negative\;control}-OD_{empty\;control}}\times 100\%$$

### 3.6. MTS-Reduction Assay

The antifungal activity of ZnFLC on the *C. albicans* HL973 were determined by the MTS assay as described in literature [26]. Briefly, the *C. albicans* HL973 were plated on 96-well plates at a density of 1.0 × 10^6^ cells/mL. After a 24 h, the dilutions of ZnFLC and FLC at different doses (4, 8, 16, 32, 64, 128, and 256 μg/mL) were added and allowed to incubate for 48 h. The *C. albicans* HL973 cells in the negative control group were treated with the same volume of medium. To evaluate cell viability, an MTS [(3-(4,5-dimethylthiazol-2-yl)-5-(3-carboxymethoxyphenyl)-2-(4-sulfophenyl)-2*H*-tetrazolium)] assay was performed according to manufacturer’s instructions (Promega, Madison, WI, USA). The cells were incubated in the dark for another 25 min at 37 °C. Then, using a multichannel pipette remove 80 μL of the resulting colored supernatant from each well and transfer into the corresponding wells of a new microtiter plate. Measured microplate absorbance at a wavelength of 490 nm on a microplate reader (Biotek Co., Winooski, VT, USA).

### 3.7. Growth Inhibition Curves

Rejuvenation of *C. albicans* HL973 was prepared in YPD (yeast peptone dextrose) liquid medium overnight at 35 °C. *C. albicans* HL973 was diluted at the starting inoculum of 1 × 10^6^ cells/mL in glass tubes. Different concentrations of the compound (8, 16, 32 μg/mL) and FLC (4.64, 9.28, 18.56 μg/mL) were added into tubes. At predetermined time points (0, 2, 4, 8, 12, 24, 36, and 48 h) after incubation in an orbital shaker (about 180 rpm) at 35 °C, a 100 μL aliquot was removed from every solution and was at 600 nm with microtiter plate reader (Thermo LabSystems Multiskan MK3), and background optical densities were subtracted from that of each well.

### 3.8. AO/EB Double Staining

living/dead staining of *C. albicans* HL973 cells with acridine orange (AO) and ethidium bromide (EB) was done as described previously with some modifications. Fungal strains were seeded at a density of 1.0 × 10^6^ cells/mL in 6-well plates. Then cells were treated with 64 μg/mL of ZnFLC and equivalent dose of FLC (58 wt/wt %) in ZnFLC for 24 h and 48 h at 37 °C. The cells were rinsed with PBS and stained with AO (100 μg/mL) and EB (10 μg/mL) for 1 min at room temperature in the dark. The cells was observed using the fluorescence microscopy (Leica DMi8, Wetzlar, Germany).

### 3.9. Assessment of Ergosterol Content

*C.albicans* HL973 were treated with 16 μg/mL of ZnFLC and equivalent dose of FLC in ZnFLC (58 wt %) at 35 °C for 24 h. The cells were centrifuged and washed with PBS. A 0.5 g wet weight of cell mixed with PBS and fresh saponifier was saponified at 80 °C for 1 h and extracted by petroleum ether. Then the extract was volatilized to dryness at 60 °C. The dry residues were dissolved by 0.5 mL methanol and filtered through 0.45 μm micro membrane. Quantification of ergosterol in samples with or without the drugs was determined by comparing peak areas of samples to a standard curve generated from HPLC-grade ergosterol. A standard curve of HPLC-grade ergosterol consisted of 0.001, 0.004, 0.015, 0.0625, and 0.25 mg/mL. Ergosterol contents were analyzed using LC-20AB prominence Liquid Chromatograph (Shimadzu Co., Kyoto, Japan) including Shimadzu C_18_ column (250 × 4.6 mm, 5 μm). Eluent was methanol/water (97/3, 100% HPLC grade). Flow rate was 1 mL/min. Temperature was 35 °C. A SPD-20AV prominence UV–vis detector (Shimadzu) was used to detect UV at 282 nm [27].

### 3.10. Real-Time PCR

Real-Time PCR was used to measure the transcriptional expressions of the genes involved in ergosterol biosynthesis of *C. albicans* HL973 treated with ZnFLC. Total RNA was extracted from *C. albicans* HL973 incubated with or without 8 μg/mL of ZnFLC and equivalent dose of FLC (58 wt/wt %) in ZnFLC for 24 h using the hot phenol method. Reverse transcription was conducted in a total volume of 20 μL with Prime script RT reagent kit. Real-time PCR reactions were performed with SYBR Green I, using qTOWER 2.0 PCR system (Analytic Jena AG, Jena, Germany). The primer sequences used in real-time PCR were listed in Table S3, using 18S rRNA as the internal control. The expression level of each gene in ZnFLC treated sample relative to that of untreated sample was calculated using 2^−^^△△Ct^ method.

## 4. Conclusions

In summary, a new compounds Zn_3_(FLC)_6_V_10_O_28_·10H_2_O exhibited potent anti-candidal effect via inducing membrane disruption. Therefore, we propose that Zn_3_(FLC)_6_V_10_O_28_·10H_2_O is a potential candidate in the development of a novel antimicrobial agent. Future research may focus on attempting to explore the novel antimicrobial POM nano-delivery system or synthesize new POM-based compounds.

## Figures and Tables

**Table 1:** Crystal data and structure refinements for ZnFLC.

Compound	ZnFLC
Formula	C_78_H_72_F_12_N_36_O_44_V_10_Zn_3_
Formula weight	3151.23
T(K)	300(2)
Crystal system, space group	Monoclinic, *P*2_1_/c
Unit cell dimensions	a = 16.983(2) Å, α = 90°
	b = 17.773(2) Å, β = 110.3 (4)°
	c = 20.033(3) Å, γ = 90°
Volume (Å^3^)	5670.3(1)
Z, ρcalcd (g cm^−3^)	2, 1.846
*μ* (mm^−1^)	1.526
F(000)	3144
Crystal size	0.32 × 0.25 × 0.21 mm
Theta range for data collection	2.29–25.08°
Limiting indices	−20 ≤ h ≤ 20, −21 ≤ k ≤ 21, −23 ≤ l ≤ 23
Reflections collected/unique	79,761/10,019 [R_int_ = 0.1083]
Completeness to θ = 25.08	99.6%
Max. and min. transmission	0.726 and 0.639
Data/restraints/parameters	10,019/0/826
Goodness-of-fit on *F*^2^	1.068
Final R indices [I > 2sigma(I)]	R_1_ = 0.0672, *w*R_2_ = 0.1777
R indices (all data)	R_1_ = 0.1043, *w*R_2_ = 0.2253
Largest diff. peak and hole	1.357 and −0.767 e Å^−3^

**Table 2:** MIC values (μg/mL) of FLC and ZnFLC against fungi. MIC values were determined according to CLSI protocol M38-A. FLC, fluconazole.

Strains	MIC_80_		MIC_50_	
	FCZ	ZnFLC	FCZ	ZnFLC
*C. albicans*				
HL973	64	4	16	2
HL963	64	32	4	1
HL996	2	4	1	0.5
HL27	2	1	1	0.5
HL3929	>256	128	>256	64
HL3973	16	8–16	8	4
HL3863	16	8	4	0.5
HL3084	16	32	4	8–16
HL3961	4	2	1	0.5
HL17034	8	16	4	4
HL3916	64	64	8	16
HL3974	16	4	0.5	0.5
HL3970	16	32	0.5	2
HL3968	32	8	4	1
ATCC 90028	1	1	0.25	0.5
*C. glabrat*				
HL981	>256	64–128	128–256	32
*C. krusei*				
HL946	>256	64–128	>256	32
*C. parapsilosis*				
ATCC 22019	2	1	1	0.5
*C. tropicalis*				
ATCC 750	4	8	4	<4

## Supplementary Materials

Figure S1: (a) and (b) Ball-stick representations of the coordination modes of Zn1 and Zn2 in ZnFLC, Figure S2: FT-IR spectrum of ZnFLC, Figure S3: The viability effects of Zn(OAc)2·H2O and NaVO3 on 9 *C. albicans* strains with the equivalent doses (wt %) in ZnFLC (MIC80) by MTX assay. Data are presented as the mean ± SD of three independent experiments, Figure S4: HPLC graphs of ergosterol in *C. albicans* HL973 treated by DMSO (a), FLC (b) and ZnFLC (c). The ergosterol extraction of DMSO, FLC and ZnFLC were diluted into 10, 10, and 1 mL with methanol. The retention time of ergosterol was about 12.9 min. Each graph displayed three repeated experiments, Table S1: Bond lengths [Å] and angles [o] for ZnFLC, Table S2: Ergosterol content of *C. albicans* HL973 treated with or without Drugs. Data are presented as the mean ± SD of three independent experiments. * p < 0.05 for FCZ and ZnFLC vs. control, ^#^ p < 0.05 for ZnFLC vs. FCZ, Table S3: Primers used for Real-Time PCR.
