# Supplementary material for: In Vitro Anticandidal Activity and Mechanism of a Polyoxovanadate Functionalized by Zn-Fluconazole Complexes
Source: Molecules. 2018 May 9;23(5):1122. doi: 10.3390/molecules23051122 (PMC6100367; doi:10.3390/molecules23051122)
Supplement: Supplementary file 1 [file molecules-23-01122-s001.pdf]

Supplementary Information

Antifungal activities and mechanism of a polyoxovanadate functionalized by Zn-fluconazole complexes

Shuanli Guo[a,†], Wei Yang[b,†], Mingming Zhao[a], RuiTian[a], Boyu Zhang[a], Yanfei Qi[a,*]

[a]School of Public Health, Jilin University, Changchun, Jilin 130021, P. R. China.

[b]College of Basic Medical Science, Jilin University, Changchun, Jilin 130021, P. R. China

Table S1 Bond lengths [Å] and angles [o] for ZnFLC.

Bonds			
V(1)-O(4)	1.601(5)	V(2)-O(5)	1.631(5)
V(1)-O(3)	1.799(5)	V(2)-O(9)	1.820(5)
V(1)-O(13)#1	1.887(5)	V(2)-O(10)	1.831(5)
V(1)-O(9)#1	1.902(5)	V(2)-O(8)#1	1.978(4)
V(1)-O(7)	2.113(5)	V(2)-O(11)	2.029(4)
V(1)-O(12)	2.288(4)	V(2)-O(12)#1	2.210(4)
V(3)-O(1)	1.597(5)	V(4)-O(6)	1.614(5)
V(3)-O(2)	2.027(5)	V(4)-O(13)	1.816(5)
V(3)-O(3)#1	1.850(5)	V(4)-O(14)	1.841(5)
V(3)-O(14)	1.856(5)	V(4)-O(11)#1	2.003(4)
V(3)-O(10)	1.904(5)	V(4)-O(8)	2.021(5)
V(3)-O(12)#1	2.325(4)	V(4)-O(12)#1	2.237(4)
V(5)-O(7)	1.687(4)	Zn(1)-OW2	1.982(6)
V(5)-O(8)	1.906(4)	Zn(1)-OW1	2.019(5)
V(5)-O(11)	1.932(4)	Zn(1)-N(1)	2.021(6)
V(5)-O(12)	2.086(4)	Zn(1)-N(13)	2.122(6)
V(5)-O(12)#1	2.181(4)	Zn(1)-OW3	2.180(6)
V(5)-O(2)	1.702(4)	Zn(2)-N(7)	2.220(6)
Zn(2)-N(7)#2	2.220(6)	Zn(2)-N(6)	2.277(6)
Zn(2)-N(10)	2.129(6)	Zn(2)-N(6)#2	2.277(6)
Zn(2)-N(10)#2	2.129(6)	F(5)-C(21)	1.333(12)
N(1)-C(2)	1.349(10)	C(4)-O(17)	1.410(8)
N(1)-C(1)	1.352(9)	C(4)-C(5)	1.514(9)

C(1)-N(3)	1.317(9)	C(4)-C(11)	1.548(9)
F(1)-C(39)	1.346(9)	F(6)-C(19)	1.340(9)
F(2)-C(8)	1.365(11)	N(4)-C(13)	1.340(9)
C(2)-N(2)	1.311(10)	N(4)-N(5)	1.364(9)
N(2)-N(3)	1.364(8)	N(4)-C(11)	1.458(9)
F(3)-C(10)	1.342(10)	C(5)-C(10)	1.383(11)
F(4)-C(37)	1.355(10)	C(5)-C(6)	1.384(10)
C(3)-N(3)	1.474(8)	N(5)-C(12)	1.296(10)
C(3)-C(4)	1.539(9)	C(6)-C(7)	1.394(11)
N(6)-C(13)	1.314(9)	C(16)-C(17)	1.550(10)
N(6)-C(12)	1.360(10)	N(16)-C(33)	1.320(11)
C(7)-C(8)	1.365(16)	N(16)-N(17)	1.357(9)
N(7)-C(14)	1.327(9)	N(16)-C(31)	1.441(10)
N(7)-C(15)	1.361(10)	O(16)-C(17)	1.406(8)
C(8)-C(9)	1.351(16)	C(17)-C(18)	1.517(10)
N(8)-C(15)	1.316(11)	C(17)-C(24)#2	1.557(9)
N(8)-N(9)	1.362(9)	N(17)-C(32)	1.319(12)
C(9)-C(10)	1.377(12)	C(18)-C(23)	1.387(11)
N(9)-C(14)	1.332(9)	C(18)-C(19)	1.388(11)
N(9)-C(16)	1.458(9)	N(18)-C(33)	1.313(12)
N(10)-C(27)	1.323(10)	N(18)-C(32)	1.349(13)
N(10)-C(28)	1.335(10)	C(19)-C(20)	1.394(12)
N(11)-C(27)	1.328(10)	C(20)-C(21)	1.351(16)
N(11)-N(12)	1.349(9)	C(21)-C(22)	1.359(15)
N(12)-C(28)	1.325(9)	C(22)-C(23)	1.412(13)
N(12)-C(29)	1.456(9)	C(24)-C(17)#2	1.557(9)
N(13)-C(25)	1.331(10)	C(29)-C(30)	1.541(10)
N(13)-C(26)	1.374(10)	C(30)-C(34)	1.527(10)
N(14)-C(26)	1.309(11)	C(30)-C(31)	1.551(10)
N(14)-N(15)	1.361(9)	C(34)-C(39)	1.371(10)
N(15)-C(25)	1.312(9)	C(34)-C(35)	1.374(10)
N(15)-C(24)	1.460(9)	C(35)-C(36)	1.393(11)
O(15)-C(30)	1.437(9)	C(36)-C(37)	1.369(13)
C(37)-C(38)	1.366(13)	C(38)-C(39)	1.374(12)
Angle			
O(4)-V(1)-O(3)	103.6(2)	O(5)-V(2)-O(9)	102.2(2)
O(4)-V(1)-O(13)#1	102.1(2)	O(5)-V(2)-O(10)	102.7(2)
O(3)-V(1)-O(13)#1	92.9(2)	O(9)-V(2)-O(10)	94.1(2)
O(4)-V(1)-O(9)#1	100.0(2)	O(5)-V(2)-O(8)#1	99.1(2)
O(3)-V(1)-O(9)#1	92.6(2)	O(9)-V(2)-O(8)#1	92.3(2)
O(13)#1-V(1)-O(9)#1	155.2(2)	O(10)-V(2)-O(8)#1	155.36(19)
O(4)-V(1)-O(7)	99.2(2)	O(5)-V(2)-O(11)	98.3(2)
O(3)-V(1)-O(7)	157.2(2)	O(9)-V(2)-O(11)	157.95(19)
O(13)#1-V(1)-O(7)	82.63(18)	O(10)-V(2)-O(11)	89.07(19)

O(9)#1-V(1)-O(7)	82.96(19)	O(8)#1-V(2)-O(11)	76.48(18)
O(4)-V(1)-O(12)	173.4(2)	O(5)-V(2)-O(12)#1	173.9(2)
O(3)-V(1)-O(12)	82.90(19)	O(9)-V(2)-O(12)#1	82.09(18)
O(13)#1-V(1)-O(12)	78.48(17)	O(10)-V(2)-O(12)#1	81.11(18)
O(9)#1-V(1)-O(12)	78.30(17)	O(8)#1-V(2)-O(12)#1	76.26(16)
O(7)-V(1)-O(12)	74.32(16)	O(11)-V(2)-O(12)#1	76.87(16)
O(1)-V(3)-O(3)#1	104.3(2)	O(6)-V(4)-O(13)	102.8(2)
O(1)-V(3)-O(14)	103.8(3)	O(6)-V(4)-O(14)	102.9(2)
O(3)#1-V(3)-O(14)	92.3(2)	O(13)-V(4)-O(14)	96.7(2)
O(1)-V(3)-O(10)	100.6(3)	O(6)-V(4)-O(11)#1	98.7(2)
O(3)#1-V(3)-O(10)	89.3(2)	O(13)-V(4)-O(11)#1	90.83(19)
O(14)-V(3)-O(10)	154.4(2)	O(14)-V(4)-O(11)#1	154.91(19)
O(1)-V(3)-O(2)	99.5(2)	O(6)-V(4)-O(8)	99.0(2)
O(3)#1-V(3)-O(2)	156.1(2)	O(13)-V(4)-O(8)	156.10(19)
O(14)-V(3)-O(2)	84.09(19)	O(14)-V(4)-O(8)	87.92(19)
O(10)-V(3)-O(2)	84.18(19)	O(11)#1-V(4)-O(8)	76.10(17)
O(1)-V(3)-O(12)#1	174.3(2)	O(6)-V(4)-O(12)#1	173.8(2)
O(3)#1-V(3)-O(12)#1	80.82(18)	O(13)-V(4)-O(12)#1	81.30(18)
O(14)-V(3)-O(12)#1	78.37(18)	O(14)-V(4)-O(12)#1	81.03(18)
O(10)-V(3)-O(12)#1	76.66(17)	O(11)#1-V(4)-O(12)#1	76.49(16)
O(2)-V(3)-O(12)#1	75.27(16)	O(8)-V(4)-O(12)#1	76.27(16)
O(7)-V(5)-O(2)	106.3(2)	O(2)-V(5)-O(12)	164.6(2)
O(7)-V(5)-O(8)	98.9(2)	O(8)-V(5)-O(12)	80.84(17)
O(2)-V(5)-O(8)	97.0(2)	O(11)-V(5)-O(12)	81.71(17)
O(7)-V(5)-O(11)	98.2(2)	O(7)-V(5)-O(12)#1	167.86(19)
O(2)-V(5)-O(11)	95.2(2)	O(2)-V(5)-O(12)#1	85.83(19)
O(8)-V(5)-O(11)	155.27(19)	O(8)-V(5)-O(12)#1	79.97(17)
O(7)-V(5)-O(12)	89.09(19)	O(11)-V(5)-O(12)#1	79.56(17)
O(12)-V(5)-O(12)#1	78.79(18)		

Symmetry transformations used to generate equivalent atoms: #1 -x+1,-y-1,-z-1; #2 -x,-y,-z

Table S2. Ergosterol content of C. albicans HL 973 treated with or without Drugs. Data are presented as the mean ± SD of three independent experiments. *P<0.05 for FCZ and ZnFLC vs. control, #P<0.05 for ZnFLC vs. FCZ

	Concentration (μg/mL)	Ergosterol content(mg/mL)
DMSO	-	7.46±0.01
FCZ	16	4.30±0.01*
ZnFLC	16	0.60±0.028* #

Table S3. Primers used for Real-Time PCR.

Gene	Primer sequence (5′-3′)	Size (bp)
18S	F:TCTTTCTTGATTTTGTGGGTGG<br>R: TCGATAGTCCCTCTAAGAAGTG	150

ERG1	F: AAGGGCAAAGGTCATGTGTT<br>R: CGTTAGCAGCAGAAGGAGGT	121
ERG7	F: TTATGCGTCGATGTTTGCAT<br>R: CCACCGTCTGGAAGTTGTTT	117
ERG11	F: TTTGACCGTTCATTTGCTCA<br>R: GCAGCATCACGTCTCCAATA	110
ERG27	F: TTGCTGCTGCTTTAGGTCAA<br>R: GTCCAGACCAGTGCTGTCAA	110
ERG28	F:GCAAGAACTTTTGGAACTTGG<br>R: TGCAGCAATAGCAAATGTGA	117

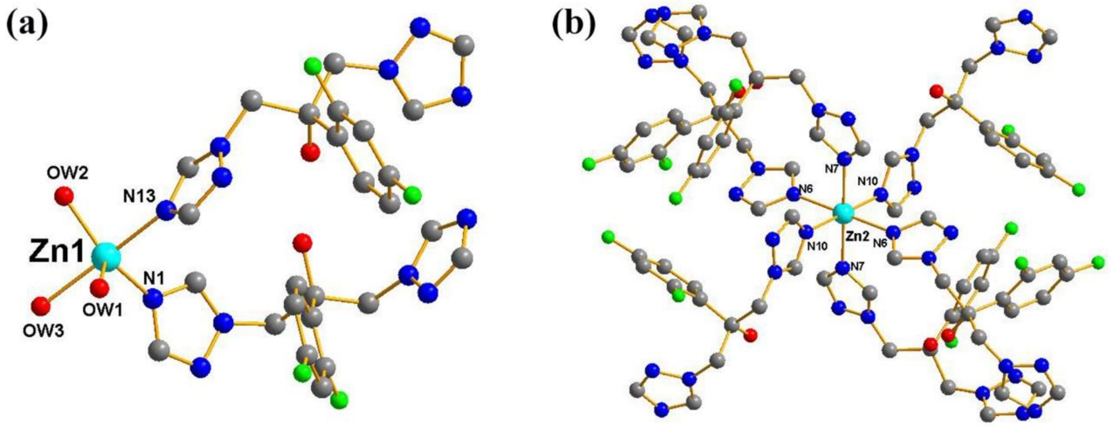

Fig. S1 (a) and (b) Ball-stick representations of the coordination modes of Zn1 and Zn2 in ZnFLC.

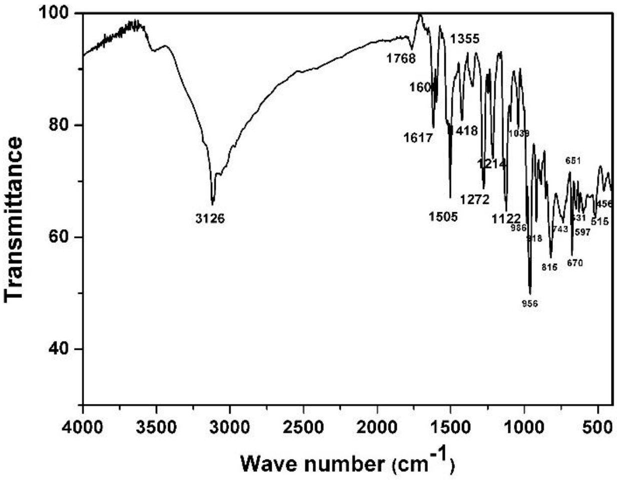

Fig. S2. FT-IR spectrum of ZnFLC.

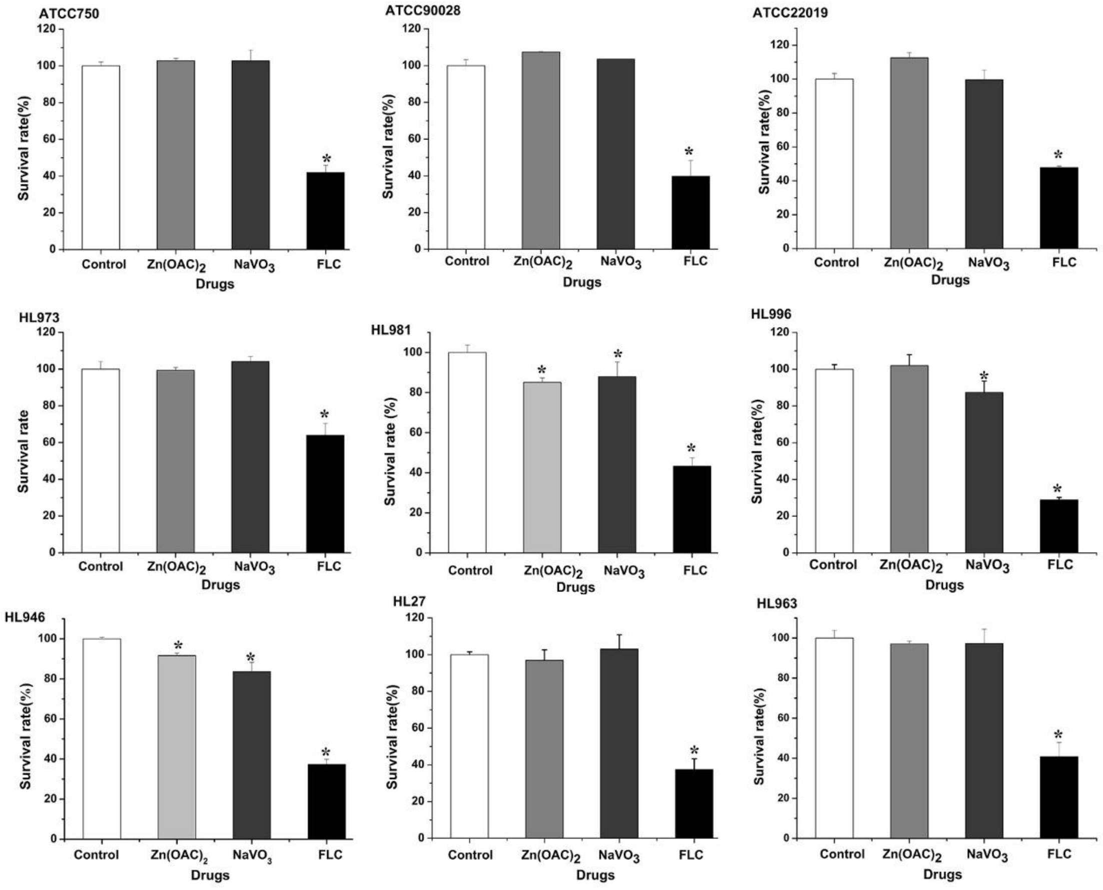

Fig. S3. The viability effects of $Zn(OAc)_2 \cdot H_2O$ and $NaVO_3$ on 9 *C. albicans* strains with the equivalent doses (%wt) in ZnFLC (MIC_{80}) by MTX assay. Data are presented as the mean ± SD of three independent experiments.

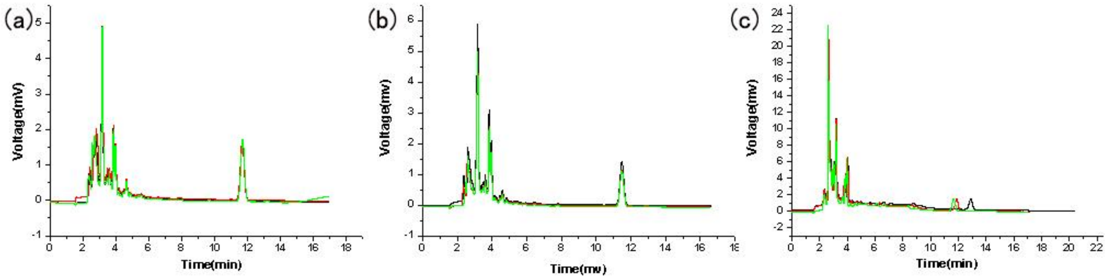

Fig. S4 HPLC graphs of ergosterol in *C. albicans* HL973 treated by DMSO (a), FLC (b) and ZnFLC (c). The ergosterol extraction of DMSO, FLC and ZnFLC were diluted into 10, 10, and 1mL with methanol. The retention time of ergosterol was about 12.9 min. Each graph displayed three repeated experiments.
